# Tuning Patchy Bonds Induced by Critical Casimir Forces

**DOI:** 10.3390/ma10111265

**Published:** 2017-11-03

**Authors:** Truc A. Nguyen, Arthur Newton, Daniela J. Kraft, Peter G. Bolhuis, Peter Schall

**Affiliations:** 1 Institute of Physics, University of Amsterdam, 1098 XH Amsterdam, The Netherlands; ngtranh@gmail.com; 2 Faculty of Electrical Engineering, Electronics-Telecommunications, Cantho University of Technology, Cantho 901184, Vietnam; 3 Van’t Hoff Institute for Molecular Sciences, University of Amsterdam, 1098 XH Amsterdam, The Netherlands; arthur.newton@gmail.com (A.N.); p.g.bolhuis@uva.nl (P.G.B.); 4 Huygens-Kamerlingh Onnes Laboratory, Leiden University, 2333 CA Leiden, The Netherlands; kraft@physics.leidenuniv.nl

**Keywords:** colloidal assembly, patchy colloid, critical Casimir effect

## Abstract

Experimental control of patchy interactions promises new routes for the assembly of complex colloidal structures, but remains challenging. Here, we investigate the role of patch width in the assembly of patchy colloidal particles assembled by critical Casimir forces. The particles are composed of a hydrophobic dumbbell with an equatorial hydrophilic polymer shell, and are synthesized to have well-defined patch-to-shell area ratios. Patch-to-patch binding is achieved in near-critical binary solvents, in which the particle interaction strength and range are controlled by the temperature-dependent solvent correlation length. Upon decreasing the patch-to-shell area ratio, we observe a pronounced change of the bonding morphology towards directed single-bonded configurations, as clearly reflected in the formation of chain-like structures. Computer simulations using an effective critical Casimir pair potential for the patches show that the morphology change results from the geometric exclusion of the increasingly thick hydrophilic particle shells. These results highlight the experimental control of patchy interactions through the engineering of the building blocks on the way towards rationally designed colloidal superstructures.

## 1. Introduction

Colloidal self-assembly, the bottom-up assembly of structures from nano- and micron-size building blocks, has recently seen major breakthroughs. Lessons learnt from colloidal self-assembly can be used to fabricate novel materials with applications in nanoscience, i.e., photonics [1,2], opto-electronics [3], and bio-materials [4]. A number of successful assembly strategies involve either controlling the interactions between the particles by use of polymer-mediated depletion interactions [5], controlled charge [6,7,8] and magnetic interactions [9,10], and DNA mediated interactions [11,12,13]; or by designing site-specific particle anisotropy with well-defined geometrical shapes and surface properties [14,15]. Indeed, recent breakthroughs in particle synthesis allow precise control of particle shape and surface affinity, offering patchy particles with specific wetting properties and symmetry [16,17]. Motivated by this experimental progress, many simulation studies have investigated the assembly behaviour of patchy particles [18,19,20,21,22,23,24,25], addressing in detail the effect of patch width, number, and geometry on the resulting topology and phase behaviour of patchy particles. Notwithstanding these simulations, experimental control in the assembly of patchy particles still remains limited. In particular, implementing the high degree of interaction control in a way similar to simulations is challenging. While model patchy interactions have been realized with DNA-coated particles providing selective bonding [13], a generic route to patchy interactions with full control over the interaction strength, range, and patch width remains challenging.

Particle interactions of tunable strength and range can be induced in binary solvents close to their phase separation temperature [26,27]. In particular, close to the solvent critical point, the critical Casimir effect allows fine control over the particle assembly determined by the temperature-dependent solvent correlation length [28]. We recently showed that by using patchy particles with hydrophobic patches and hydrophilic equatorial shells, we can realize selective patch-to-patch binding with tunable strength and range [29]. The critical Casimir force results from the confinement of solvent fluctuations between the particle surfaces in the near-critical mixture [30,31], and because it depends on the wetting boundary conditions, it offers selective bonding of hydrophobic or hydrophilic patches. The advantage is that the interaction range and strength is set by the solvent correlation length that adjusts with temperature in a universal manner. This universal, in situ control of particle interactions offers new opportunities to assemble patchy particles in and out of equilibrium, and to anneal structures once they are formed. Moreover, by using particles with well-defined patch sizes, this allows us to experimentally explore the effect of patch size on the assembled structures.

In this paper, we experimentally investigate the effect of patch width on the bonding topology of patchy particles assembled by critical Casimir forces. Using particles synthesized with well-defined patch-to-shell area ratios in near-critical binary solvents, we achieve control over both the width and strength of the patchy particle interaction. We show that decreasing the patch area fraction towards narrow patches tailors the bonding to change from multi-particle bonding, where more than two patches bind together, to single bonding, where only one patch binds to another one. Using dimer particles as an example, we demonstrate that as a result, the topology of colloidal aggregates becomes much more distinct, clearly favoring directed chain-like structures. Computer simulations on patchy particles with shape and area ratios taken from the experiment and interacting with an effective critical Casimir pair potential model are in good agreement with the experimentally observed topologies, and show that this morphology change is due to geometric exclusion of the bonding particles. These qualitative results demonstrate the experimental control in tuning the assembly behaviour via the patchy particle design, offering a new generic approach to the rational design of colloidal superstructures.

## 2. Experiment

**Figure 1:**
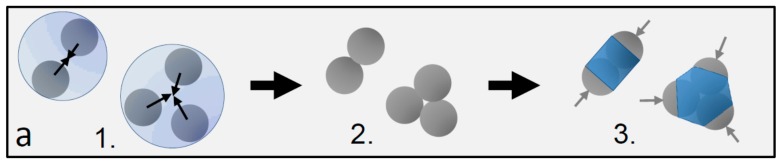

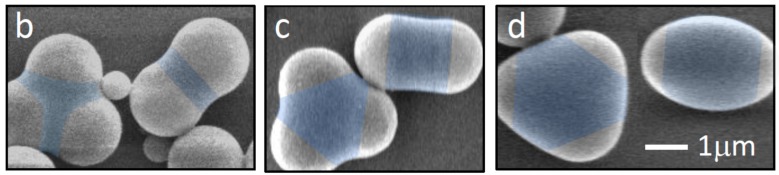
Dimer and trimer particles with varying patch-to-shell area ratios. (**a**) Schematic of the particle preparation process yielding a mixture of dimers, trimers, and higher-order patchy particles: 1. particle clustering in emulsion droplets, 2. resulting particle clusters, and 3. final patchy particles after the growth of the polymer shell. Gray hemispheres (arrows) demarcate the patches, and the blue central part demarcates the shell; (**b**–**d**) Scanning electron microscopy images of dimer and trimer patchy particles obtained with 0.1 mL (**a**), 0.2 mL (**b**), and 0.4 mL monomer mixture (**c**) added during the swelling process. Blue colored areas indicate the estimated shell, and light gray areas indicate the estimated patch size. The shell grows and the patch shrinks with increasing amounts of monomer mixture from left to right.

**Figure 2:**
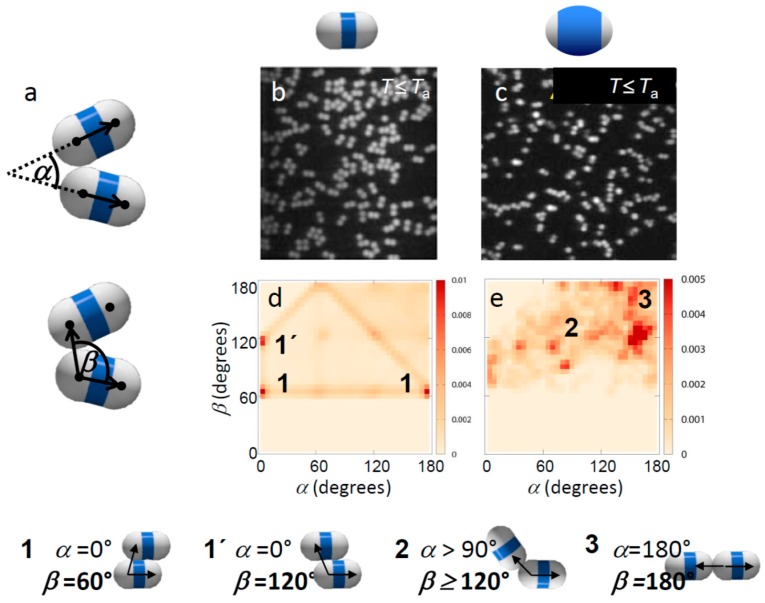
Effect of patch width on weak critical Casimir bonding. (**a**) Definition of bond angles *α* and *β*; (**b**,**c**) Confocal microscope images of weakly bonded dimer particles with thin (**b**) and thick shells (**c**). The temperature was set to just below *T*_a_, where bonds are weak and break frequently, with an attractive strength of the patches (total potential minimum) of ~3*k*_B_*T*, as estimated from combined modeling and pair correlation measurements [37]. (**d**,**e**) Map showing *α*–*β* angle probability distributions of weakly bonded thin (**d**) and thick-shell particles (**e**). Frequently observed configurations are labelled and illustrated below. Bottom: Illustration of typical bonding configurations of dimer particles. The bond angle *β* is visualized by arrows.

Our multivalent particles are made by swelling and polymerizing clusters of polymethylmethacrylate (PMMA) spheres of radius *R* = 1.15 μm with a methylmethacrylate/methacrylic acid shell, resulting in geometrically well-defined patches with rotational symmetry [14], as shown schematically in Figure 1a. We use fluorescently labelled 4-methylaminoethylmethacrylate-7-nitrobenzo-2-oxa-1,3-diazol (NBD-MAEM) PMMA spheres with grafted poly(12-hydroxystearic acid) copolymer (PHS-g-PMMA) as steric stabilizer as suggested by Elsesser et al. [32]. Colloidal clusters are fabricated by encapsulating the spherical PMMA particles in toluene droplets in an aqueous phase and selectively evaporating the toluene (anhydrous, 99.8%, Sigma-Aldrich, St. Louis, MO, USA); see Figure 1a, steps 1 and 2 [14]. To obtain patchy particles, we grew PMMA shells around the colloidal clusters; see Figure 1a, step 3. By varying the amount of monomer during the growth of the shells, we achieved particles with different, well-defined patch-to-matrix area ratios. Specifically, the shells were grown using a suspension of 0.5 mL PMMA clusters (2% *w/w*), 0.2 mL 1% *w/w* sodium dodecyl sulfate, 0.3 mL water, and varying amounts of a monomer mixture consisting of 97:2:1 *w/w* methylmethacrylate:methacrylic acid:ethylene glycol dimethacrylate: 0.1, 0.2, and 0.4 mL. The suspension was stirred in a 5 mL round bottom flask with a Teflon-coated magnetic stir bar for 30 min. We then polymerized the swollen particles in an oil bath at 80 °C for 6 h by the addition of 350 μL 7.4 mM aqueous potassium persulfate (99.99%, Sigma-Aldrich). The potassium persulfate imparted sulfate charges to the PMMA shells, rendering them hydrophilic with a low surface charge density. The surface of the patches not covered by shells was equivalent to that of the PMMA spheres, whose steric stabilization by PHS-g-PMMA makes them hydrophobic. The sample was purified by washing three times with deionized water and re-dispersed by adding water with 1% *w/w* sodium dodecyl sulphate (≥99.0%, Sigma-Aldrich). This synthesis route yields a mixture of monomer, dimer, trimer, and higher-symmetry particles. To separate these particle types, we employed density gradient centrifugation using a sucrose gradient in water. By extracting the second and third bands using a syringe, we obtained a high purity of dimer and trimer particles, respectively. The different amounts of monomer added during the swelling process resulted in different thicknesses of the shells: a larger amount of monomer resulted in thicker shells, as shown in Figure 1b–d. To improve the site-specific properties of the particles, we washed them in an acid solution of 10 μL 0.01 M HCl in 1 mL pure water, in which we left the particles for two days. We then washed the particles three times in pure water before suspending them in the final binary solvent mixture of heavy water and 3-methylpyridine (3MP) (≥99.5%, Sigma-Aldrich). We prepared suspensions in binary solvents with 3MP weight fraction of *c*_3MP_ = 0.25 and 0.31, on the left and right side of the solvent critical point *c*_c_ = 0.28 [33]. These compositions yielded roughly the largest critical Casimir force between hydrophobic and hydrophilic surfaces, respectively, at a given near-critical temperature [34]. We added a small amount of salt (sodium chloride, 0.62 mM) to the suspension to screen the repulsive particle charges and increase the temperature interval of aggregation [35]. The final suspensions were prepared at a colloid volume fraction of 0.2% and filled into glass capillaries, which were subsequently flame sealed to prevent compositional changes associated with evaporation. As in previous studies, we worked at temperatures below the phase-separation temperature where the two solvent components, 3MP and water, form a homogenous mixture. To induce critical Casimir interactions, we heated the suspension to temperatures *ΔT* = *T*_c_ − *T* below the critical temperature *T*_c_ ~ 41.0 °C. Particles aggregate at temperatures above the aggregation temperature *T*_a_ ~ 40.05 °C, i.e., *ΔT*_a_ ~ 0.95 °C below *T*_c_. To achieve well-controlled assembly, we first equilibrated the suspension at 3 K below *T*_a_, where critical Casimir forces are still negligible and the particles remain suspended. Since the density of the particles was not matched with that of the binary solvent, the particles formed a sediment at the bottom of the sample. We allowed all the particles to sediment for at least 30 min, resulting in a quasi-2D system, before we raised the temperature to the final desired value at or above *T*_a_. After 15 more minutes, we recorded 200 images of particle configurations using confocal microscopy. In these images, the fluorescently labelled spheres of the dimer particle appear as bright dots on a dark background. We located the centers of these dots with an accuracy of 20 nm in the horizontal plane, and connected pairs of closest centers with a separation of less than 2.3*R* into dimer particles. The connecting vector indicates the orientation of the dimer particle. We used these vectors to define bond angles between adjacent bonded dimer particles. We identified bonded dimer particles as those that had sphere centers closer than 3.0*R*. Two angles were used to define the local bonding topology, see Figure 2a,b. The angle *α* defines the respective orientation of the bonded particles as the angle between the long axis vectors of the dimers. The angle *β* defines the position of the bond as the angle between the connecting vector of the bonded spheres and the long axis vector of one of the bonded dimers. With this definition, linear, bonded chains have angles *α* ~ 180° (or 0°) and *β* ~ 180° (or 0°), while side-by-side bonded particles have angles *α* ~ 0° (or 180°) and 60° < *β* < 120°.

## 3. Simulation

Similar to the experimental dimer particles, we constructed anisotropic patchy particles from two fused (tangent) spherical particles of radius *R* (forming a dumbbell) and one hard-core shell of varying radii located at the center of mass of the dumbbell. The spherical particles of the dumbbell interacted with those of a neighboring dumbbell via the potential *u*(*r*) = *u*_rep_(*r*) + *u*_att_(*r*), determined by the balance of electrostatic repulsion and critical Casimir attraction [35,36,37]. Here, *u*_rep_ = *A*_rep_exp[−(*r* − 2*R*)/*l*_D_] and *u*_attr_ = *A*_Cas_exp[−(*r* − 2*R*)/*ξ*], with *r* being the separation of the centers of two spheres not belonging to the same dumbbell, *A_el_* = (2*πRσ_c_*^2^*l_D_*^2^)/(*εε*_0_) the strength of the electrostatic repulsion, *A*_Cas_ the amplitude of the critical Casimir force and the solvent correlation length *ξ* = *ξ*(*c*_3MP_, *ΔT*) that depends on both solvent composition and temperature. The central sphere representing the hydrophilic shell was modeled by a hard-core repulsive particle interacting via *u*_rep_(*r*) with all other spheres. For our solvents with compositions close to the critical composition, we applied the simplified scaling *ξ* = *ξ*_0_(*ΔT*/*T*_c_)^−0.63^ of the critical composition, which for the temperatures applied here were within 10% (at *T*_a_) and 20% (at 0.6*ΔT*_a_) of the actual off-critical correlation length [34]. We measured the surface charge *σ*_C_ = −0.188 μC/cm^2^ by electrophoresis, and the Debye screening length *l*_D_ = 24 nm by conductivity measurements; the only free parameters were then the amplitude *A*_Cas_ and correlation length *ξ*_0_ far away from the critical point. As shown in Reference [37], we obtained good fits of experimentally measured pair correlation functions with *ξ*_0_ = 1.6 nm, larger than the typical literature values of around 0.3 nm [38], but in agreement with a recent value of 1.5 nm used in the modeling of critical Casimir potentials [39], and *A*_Cas_ = 2*πR*/*ξ*, the amplitude at the critical composition. The potential was thus optimized to reproduce the right temperature dependence of the experimental pair correlation function [37]. While the patchy particle as a whole is anisotropic and interacts via an orientation-dependent potential, the simple model was constructed as a colloidal molecule in which the different parts of the composite particle (molecule) interact isotropically. With this model, we performed Monte Carlo simulations using translation, orientation, and cluster moves. The particles were put in a cubic box and simulated under gravity, with a gravitational length based on the experimental density mismatch, to obtain a quasi-2D system. For consistency, the simulation data were analyzed in exactly the same way as the experimental data.

## 4. Results and Discussion

Suspended in the binary solvent, the particles exhibited specific binding with temperature-dependent strength and range when the suspension was heated close to *T*_c_ [34]. Specifically, we found that in 3MP-poor solvents, the hydrophobic patches attract, while in 3MP-rich solvents the shells attract [29], consistent with the fact that aggregation occurs between surfaces favoring the minority component of the solvent. Here we investigated the effect of patch width and temperature (interaction strength) on the bonding topology. In particular, one of the major challenges is to achieve single-bonded structures with a narrow bond-angle range, to create the most distinct structures. To investigate the influence of particle patch width on the bonding morphology, we focused on particles with small and large shells (Figure 1a,c). We observed that already at temperatures below *T*_a_, where particles interact weakly and no permanent bonds form, the interaction of thin-shell and thick-shell particles is very different, as seen in the representative microscope images in Figure 2b,c. Particles with thin shells showed more ambiguous configurations with many side-by-side bonded configurations (Figure 2b), while particles with thick shells showed predominantly linear, chain-like configurations (Figure 2c). This was confirmed when we plotted the distribution of bond angles *α* and *β* as shown in Figure 2d,e. In these figures, the probability of a configuration with angle *α* and *β* is indicated in a shade of red. Frequently observed configurations are demarcated in dark red. Thin-shell particles (Figure 2d) show an abundance of bond angles *α* ~ 0° and *β* ~ 60° or 120°, indicating side-by-side bonded configurations. In contrast, for thick shells, the distribution is clearly shifted towards higher values of *α*, reflecting more linear, patch-to-patch bonded configurations. Yet, due to the low attraction, the distribution is still spread and no clear peak is observed at *α*, *β* ~ 180°, which would indicate linear bonded chains.

**Figure 3:**
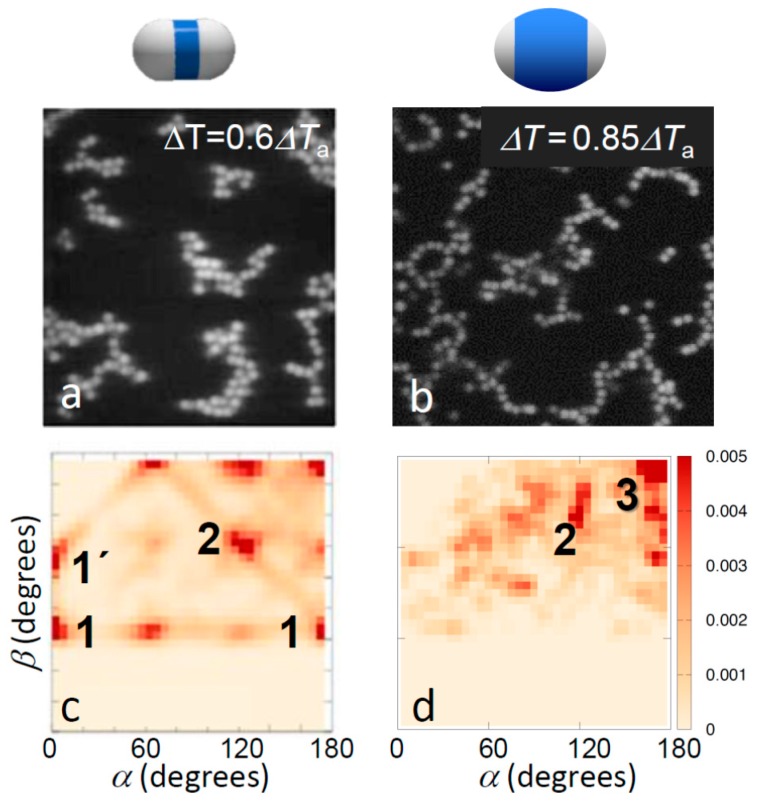
Effect of patch width on aggregate morphology for strong bonding. Confocal microscope images (**a**,**b**) and corresponding distribution of bond angles (**c**,**d**) for strongly bonded thin-shell (left) and thick-shell particles (right). The temperatures 0.6*ΔT*_a_ and 0.85*ΔT*_a_ correspond to solvent correlation length *ξ* = 15 nm and *ξ* = 10 nm, respectively, resulting in a total potential minimum *u*_min_ = −6.5*k*_B_*T* with attractive critical Casimir contribution *u*_att_(*r*_min_) = −9.2*k*_B_*T* (**a**,**c**), and *u*_min_ = −4.1*k*_B_*T* with *u*_att_(*r*_min_) = −6.3*k*_B_*T* (**b**,**d**) [37]. Clear change in the bonding morphology from close-packed to head-to-head bonding was observed. The most frequently observed bonding configurations are labelled; see bottom of Figure 2 for illustrations.

This situation changes for temperatures above *T*_a_ where due to a stronger Casimir attraction the topologies of bonded structures become more pronounced. Representative images of the aggregate structures above *T*_a_ are shown in Figure 3a,b. Clearly, particles with small shells exhibit significant bonding ambiguity: the aggregates show more compact structures, branch points, and a variety of bond angles. In contrast, particles with large shells show chain-like structures, clearly favoring single, directed bonding. This difference was again confirmed in the bond angle distributions shown in Figure 3c,d, revealing very different favored local configurations. Particles with thin shells favored a variety of configurations including side-by-side bonding (1 and 1’) and inclined head-to-head bonding (2). In fact, the regular peaks at *α*, *β* ~ 0°, 60° and 120° suggest the emergence of a lattice structure, as also reflected in the real-space image in Figure 3c, and consistent with the triangular lattice structure expected for dense two-dimensional hard-dumbbell packings [40,41]. In contrast, particles with thick shells showed a strong tendency towards linear bonded structures, as shown by the distinct peak at *α* ~ 180° and *β* ~ 180° (3), though some other configurations at lower *α*, *β* were also observed, corresponding to, for instance, bends and branches, as seen in the microscope image above. Yet, the pronounced trend was the shift of the distribution to the upper right corner of the *α*, *β* plane, indicating linear, single-bonded configurations. We thus concluded that the thinner shells and concomitant larger patches offer more room to bond and thus allow a larger range of bond angles and multiple patches to bind with each other, resulting in both sideways and inclined head-to-head bonded configurations, which ultimately yield the close-packed structures abundant in the microscope image (Figure 3c). The smaller patches resulting from the thicker shells, in contrast, leave less room to bond, narrowing down bond angles, and favoring single-bonded configurations.

**Figure 4:**
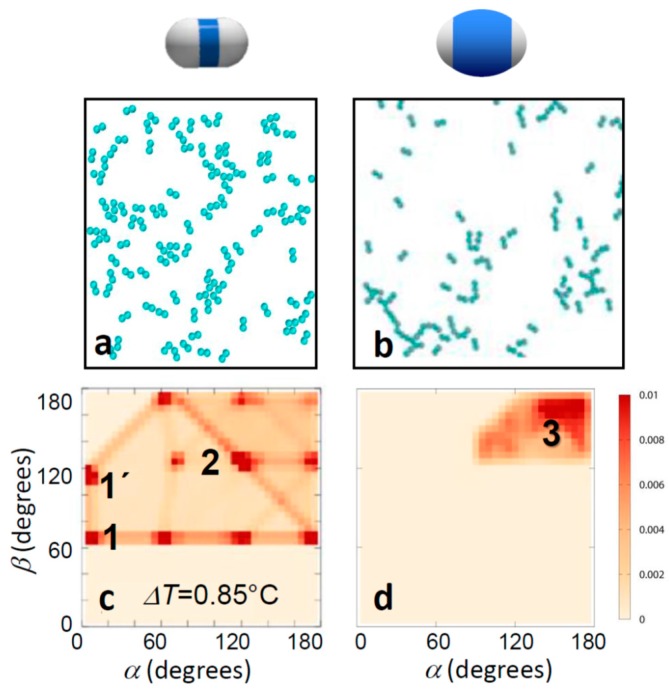
Computer simulations of bonding morphology. Snapshots of computer simulations (**a**,**b**) and corresponding bond angle distributions (**c**,**d**) of thin (left) and thick-shell particles (right). Temperatures correspond to the experimental temperatures in Figure 3a,b. The experimentally observed narrowing of bond angles towards head-to-head single bonding is clearly reproduced. This narrowing of bond angles is a direct consequence of the geometric exclusion due to the thicker shells.

These conclusions were supported by Monte Carlo simulations of dimer particles interacting via effective pair potentials, where the area ratio between the particle patches and shell was taken into account by tuning the hard-core shell to that of the experimental particles (see Simulation section above and Reference [37]). A comparison of simulated thin and thick-shell particles is shown in Figure 4. The characteristic change in the bonding morphology is clearly seen, supported by the angle distributions, which qualitatively resemble the experimental ones well. In particular, the qualitative change from the bonding ambiguity of several distinct bonding configurations for thin shells to straight head-to-head bonded configurations for thick shells was well reproduced. Again, thin-shell particles showed an abundance of side-by-side bonded (red blobs 1 and 1’) and inclined head-to-head bonded configurations (red blob 2), as well as configurations interpolating in between (red connecting lines). For thick shells, the distribution shifts to the upper right corner: side-by-side bonded configurations vanished, and those with *α*, *β* close to 180° emerged (red blob 3). As the only difference in the simulations was the presence of a differently sized hard-core, we concluded that a simple geometric exclusion is enough to cause the change in bonding topology, and for thick enough shells, it is sufficient to achieve single bonded configurations.

These results are also in line with simulations based on other dimer patchy particle models, consisting of a single sphere with opposing patchy caps [23]. Varying the width of the patchy caps, the authors observed that for patches sufficiently small so that a patch can only bind with one other patch (surface coverage below ~6% of the total sphere surface), the simulated particles formed chain-like structures, while for larger patches allowing two or more patchy bonds, the particles formed close-packed configurations, assembling into close-packed planes. We note, however, that for critical Casimir forces, many-body interactions [42] and synchronization phenomena [43] can lead to additional effects not accounted for in the simulations based on pairwise additive potentials; such effects are expected to be particularly pronounced close to *T*_c_, where they can influence the transition from multi-particle bonding to chain-like structures.

**Figure 5:**
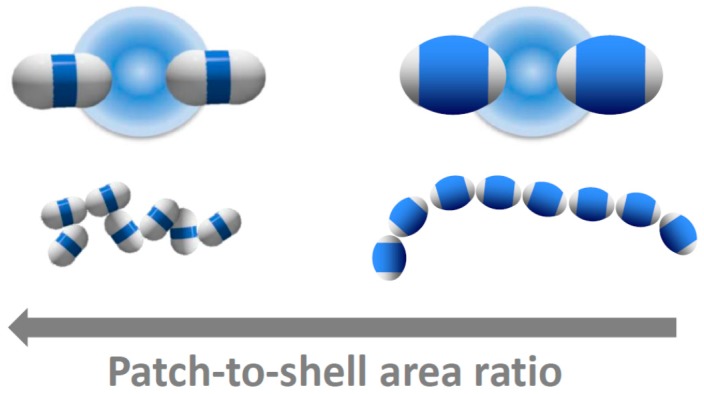
Tuning patchy critical Casimir bonds with patch size. Schematic of the bonding of dimer particles with large (**left**) and small attractive patches (**right**). Top: Artist’s impression of critical Casimir bonding of patchy particles. Bottom: Representative particle configurations reconstructed from confocal microscope images of bonded particles. The hydrophobic patches attract in solvents with composition *c*_3MP_ < *c*_c_. Decreasing the patch-to-shell area ratio results in increasingly chain-like, linear structures.

The dimer patch-to-shell area ratio therefore drastically affects the bonding configuration. We summarize our results in Figure 5. A large patch-to-shell ratio leads to a large range of bond angles and bond positions, resulting in a wide variety of bonding configurations (left). A small patch-to-shell ratio leads to a much narrower range of bond angles and binding sites, resulting in much more distinct, chain-like structures (right). These results provide guideline for particle design in the experiments. The thick-shell particles employed in this work provide a good step in the direction towards distinct single-bonded structures, and it will be interesting to see which structures result from higher-symmetries, such as trimers, tetramers, and higher-order particles and mixtures thereof.

## 5. Conclusions

By employing critical Casimir forces between patchy particles in near-critical binary solvents, we qualitatively investigated the influence of patch size on the bonding topology of dimer particles and the structure of the resulting aggregates. Selective bonding of the particle patches was achieved by grafting poly-hydroxystearic acid onto the patch surfaces, rendering them hydrophobic, and suspending these particles in 3MP-poor solvents to induce critical Casimir interactions between the patches only. Using dimer particles with varying patch-to-shell ratios, we clearly observed that a large patch-to-shell ratio leads to a large variety of bond angles and bond configurations that, in turn, yield a wide variety of aggregate structures from chains to closely packed particles. A small patch-to-shell ratio, in contrast, narrows down bond angles towards distinct linear configurations, yielding chain-like aggregates. Computer simulations indicate that this morphology change is due to the simple geometric exclusion of the hard shells. These qualitative results provide a proof-of-principle demonstration of the structural control resulting from the combination of critical Casimir forces and well-designed patchy particle building blocks. As the critical Casimir interaction is based on a universal effect, many material and solvent systems can be used, and assembly in more environmental-friendly solvent systems has been recently demonstrated [44].
